# Phase modulation of insulin pulses enhances glucose regulation and enables inter-islet synchronization

**DOI:** 10.1371/journal.pone.0172901

**Published:** 2017-02-24

**Authors:** Boah Lee, Taegeun Song, Kayoung Lee, Jaeyoon Kim, Seungmin Han, Per-Olof Berggren, Sung Ho Ryu, Junghyo Jo

**Affiliations:** 1 Department of Life Sciences, Pohang University of Science and Technology, Pohang, Gyeongbuk, Korea; 2 Asia Pacific Center for Theoretical Physics, Pohang, Gyeongbuk, Korea; 3 School of Interdisciplinary Bioscience and Bioengineering, Pohang University of Science and Technology, Pohang, Gyeongbuk, Korea; 4 The Rolf Luft Research Center for Diabetes and Endocrinology, Karolinska Institute, Stockholm, Sweden; 5 Department of Physics, Pohang University of Science and Technology, Pohang, Gyeongbuk, Korea; La Jolla Institute for Allergy and Immunology, UNITED STATES

## Abstract

Insulin is secreted in a pulsatile manner from multiple micro-organs called the islets of Langerhans. The amplitude and phase (shape) of insulin secretion are modulated by numerous factors including glucose. The role of phase modulation in glucose homeostasis is not well understood compared to the obvious contribution of amplitude modulation. In the present study, we measured Ca^2+^ oscillations in islets as a proxy for insulin pulses, and we observed their frequency and shape changes under constant/alternating glucose stimuli. Here we asked how the phase modulation of insulin pulses contributes to glucose regulation. To directly answer this question, we developed a phenomenological oscillator model that drastically simplifies insulin secretion, but precisely incorporates the observed phase modulation of insulin pulses in response to glucose stimuli. Then, we mathematically modeled how insulin pulses regulate the glucose concentration in the body. The model of insulin oscillation and glucose regulation describes the glucose-insulin feedback loop. The data-based model demonstrates that the existence of phase modulation narrows the range within which the glucose concentration is maintained through the suppression/enhancement of insulin secretion in conjunction with the amplitude modulation of this secretion. The phase modulation is the response of islets to glucose perturbations. When multiple islets are exposed to the same glucose stimuli, they can be entrained to generate synchronous insulin pulses. Thus, we conclude that the phase modulation of insulin pulses is essential for glucose regulation and inter-islet synchronization.

## Introduction

Hormones are long-distance messengers that regulate numerous body functions, including metabolism. Many hormones are secreted in an oscillatory manner [1]. Insulin, one of the most important hormones in human physiology, is also secreted in a pulsatile manner [2]. In particular, pulsatile insulin secretion or Ca^2+^ oscillation, with a period of a few minutes, has been widely observed in β cells [3], islets [4, 5], the pancreas [6], and blood [7]. Compared to constant insulin action, pulsatile insulin secretion suppresses hepatic glucose production more effectively [8], and it may prevent the desensitization of insulin receptors [9, 10]. Moreover, the pulsatility of insulin secretion is lower in diabetic patients [11] and in islet cell antibody positive non-diabetic subjects [12]. This evidence demonstrates the physiological importance of insulin pulsatility. However, we still lack a mechanistic understanding of how insulin pulses are generated and modulated in response to glucose perturbations. The period (~5 min) of insulin pulses is not sensitive to the glucose concentration, but closer review indicates that the pulse has a U-shape with the shortest period occurring at ~11 mM glucose [13]. The duration of peak/valley or active/silent phases of insulin pulses also depends on the glucose concentration. These observations imply that insulin pulses encode hormonal information in their phase and amplitude.

The islets of Langerhans generate insulin pulses. Humans have approximately one million islets in the pancreas. If insulin secretion is not pulsatile and is merely regulated by its amplitude, coordination between islets becomes unnecessary. However, the independence/coherence of pulsatile insulin secretion must have great physiological effects. It has been implicitly assumed that islets are synchronized to generate coherent insulin pulses. Otherwise, the independent pulses from the one million islets would show a flat insulin profile in the blood. Nevertheless, inter-islet synchronization has not been demonstrated directly through *in vivo* experiments. Two hypotheses, which are not mutually exclusive, have been proposed to explain inter-islet synchronization. One hypothesis is that islets, which experience a common glucose stimulus, are entrained by a rhythmic change in glucose concentration [14–22]. The other hypothesis is that islets, which are innervated by central nerves, are synchronized directly by neural signals [23, 24].

In this study, we investigate the phase modulation of insulin pulses and its functional role in glucose regulation and inter-islet synchronization. We experimentally probe how islets generate Ca^2+^ oscillations, a proxy of insulin pulses, in response to glucose stimuli. To study the entire process of glucose-insulin regulation, one needs to incorporate (i) insulin secretion by glucose and (ii) glucose regulation by insulin. Because noninvasive monitoring of insulin secretion from multiple islets regulated by glucose *in vivo* is not feasible, we developed a mathematical model that describes the glucose-insulin loop. The data-based model demonstrated that the glucose-dependent shape modulation of insulin pulses contributed to tighter regulation of the normal glucose level, compared to their amplitude modulation alone. Furthermore, the shape modulation provided a common cue for physically-separate islets to generate synchronous insulin pulses. Then the inter-islet synchronization through the glucose entrainment could enhance the pulsatility of circulating insulin levels.

## Results

### Pancreatic islets generate glucose-dependent Ca^2+^ oscillations

**Fig 1:**
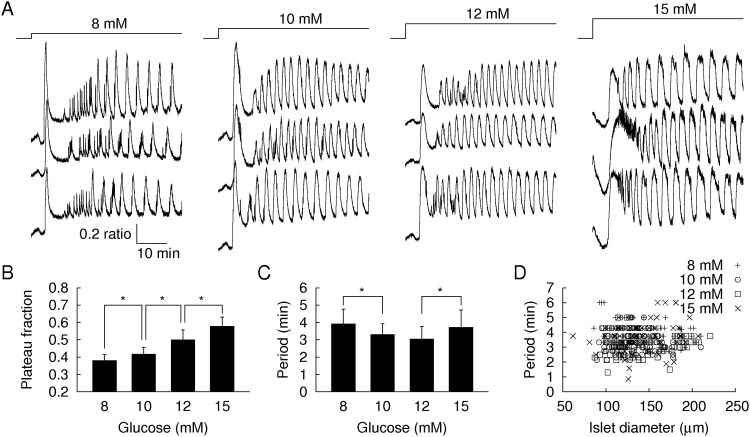
Glucose-dependent Ca^2+^ oscillations. (A) Time traces of the intracellular Ca^2+^ concentrations (340/380 nm fluorescence ratio) within each islet for 8, 10, 12, and 15 mM glucose concentrations and stimulation from the basal concentration of 3 mM. (B) Plateau fractions, ratio of active to silent phases, and (C) dominant periods of the Ca^2+^ oscillations (n = 99, 94, 64, and 64 islets for 8, 10, 12, and 15 mM glucose, respectively). Error bars represent standard deviations. *P<0.01. (D) Dominant periods versus islet diameters.

Ca^2+^ ions trigger insulin secretion from β cells [25–27]. Thus, measurements of the intracellular Ca^2+^ concentration of β cells can represent their insulin secretion. The intracellular Ca^2+^ concentration oscillates under high glucose concentrations (>7 mM). In the present study, we focused on the “slow” Ca^2+^ oscillation with a period of a few minutes. It has been suggested that autonomous oscillation originates from glycolytic oscillations [28, 29]. We measured the Ca^2+^ oscillations in islets, β cell dominant micro-organs, under high glucose concentrations of 8, 10, 12, or 15 mM (Fig 1A). When the concentration of the glucose solutions abruptly changed from 3 mM to the higher levels, the islets initially generated acute Ca^2+^ pulses, followed by stationary Ca^2+^ oscillations after some equilibration time. We collected a large amount of Ca^2+^ oscillation data (60–100 samples for each condition), which were sufficient to probe the glucose-dependent modulations. Here we excluded two samples (from total 66 samples) for 15 mM that showed prolonged active phases without regular oscillations. A recent study has reported that 15 mM is a bistable glucose concentration that can generate both oscillatory and stationary Ca^2+^ dynamics [30]. First, we examined the duration of the active and silent phases of the Ca^2+^ oscillations (See Materials and methods). We defined the ratio of active to silent phases as the “plateau fraction” [13]. The islets generated higher plateau fractions at higher glucose concentrations (Fig 1B). Second, we quantified the period of Ca^2+^ oscillations using a Fourier transformation of their time traces. Dominant periods showed a U-shaped glucose dependence (Fig 1C). Compared to 8 mM (4.1±1.1 min) and 15 mM glucose (3.8±0.9 min), 10 mM and 12 mM glucose showed shorter periods (3.3±0.6 and 2.9±0.7 min, respectively; P<0.01). We also noticed longer dominant periods accompanied by larger variations. The dominant periods were not dependent on islet size (Fig 1D).

**Fig 2:**
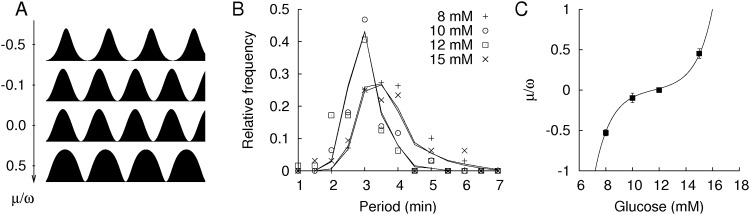
Phase modulation and periods of Ca^2+^ oscillations. (A) Phase modulated oscillations of the active rotator model. (B) Distributions of the dominant periods of Ca^2+^ oscillations under 8 mM (‘+’ symbol, n = 99), 10 mM (circle, n = 94), 12 mM (square, n = 64), and 15 mM (‘×’ symbol, n = 64) glucose. The symbols represent data, and the solid lines represent the model predictions. The predicted distributions are too close to distinguish for 8 mM and 15 mM glucose and for 10 mM and 12 mM glucose. (C) Phase modulation depending on glucose concentrations. The symbols represent estimated values and their standard deviations, and the solid line represents the nonlinear function *μ*(*G*).

We observed that these nontrivial features of the plateau factions and dominant periods could be simply described by the “active rotator model,” which has been widely used to understand oscillatory phenomena in physics [31–33]. Suppose that the spontaneous Ca^2+^ oscillations can be approximated as a periodic function,

Ca(t)=r[1+cosθ(t)], (1)

where *r* and *θ* are the amplitude and phase at time *t*, respectively, and unity is added to prevent a negative Ca^2+^ concentration. For the moment, we ignore amplitude by setting *r* = 1 to focus on the phase of the Ca^2+^ oscillations. The phases *θ* = 0 and *π* represent the peak and valley of the Ca^2+^ oscillations, respectively. If the phase linearly increases with a constant angular velocity (*dθ* / *dt = ω*), the Ca^2+^ oscillations become a pure sinusoidal wave with equal durations for the active and silent phases. However, the islet Ca^2+^ concentrations oscillate in phase-modulated waves that are dependent on the glucose concentration. The modulation can be captured by the active rotator model,

$$\frac{d\theta }{dt}=\omega -\mu \text{cos}\theta ,$$ (2)

where the strength *μ* of the pinning force controls the degree of phase modulation by breaking the rotational symmetry and forcing oscillators to remain in the active phase (*θ* = 0) for a longer time and in the silent phase (*θ* = *π*) for a shorter time for positive *μ* and vice versa for negative *μ*. In the presence of the pinning force, the effective period of the active rotator is $2\pi /\sqrt{\omega^{2}-\mu^{2}}$. Thus, the pinning force always makes the rotator slower regardless of its sign. These features of the active rotator model are consistent with the glucose-dependent plateau fraction and dominant period of the islet Ca^2+^ oscillations (Fig 2A). Thus, we hypothesize that islets have an intrinsic period in their Ca^2+^ oscillations, but the period is modified by phase modulations that are dependent on the glucose concentration. Specifically, the phase modulation was absent at 12 mM glucose because in this condition, the durations of the active and silent phases were equal (Fig 1B). Furthermore, 8 mM glucose could impose a negative *μ* that increased the silent phase and the period of Ca^2+^ oscillations, whereas 15 mM glucose could impose a positive *μ* that increased the active phase and the period of Ca^2+^ oscillations. We quantified the *μ* values to fit the Ca^2+^ oscillations under different glucose concentrations. One practical issue with the estimation is that Ca^2+^ oscillations have heterogeneous periods between islets, even for the same glucose concentration (Fig 2B). Therefore, we considered a distribution of intrinsic periods. For simplicity, we assumed that the periods follow a Gaussian distribution with a mean ($\bar{\omega }$) and standard deviation (*δω*) Thus, different glucose concentrations have different *μ* values that modulate the Gaussian distribution. With the maximum likelihood estimation of the parameters, our hypothesis could fit the period distributions of the measured Ca^2+^ oscillations (Fig 2B). The estimated *μ* values for glucose concentration (*G*) could be fit with a nonlinear function,

$$\mu (G)=\bar{\mu }\text{sinh}\left(\frac{G-G_{0}^{\mu }}{\delta G^{\mu }}\right)$$ (3)

with parameters $\bar{\mu }=0.016$ min^-1^, $G_{0}^{\mu }=11.6$ mM and *δG*^*μ*^ = 1.2 mM (Fig 2C). The phase modulation was gradual at ~12 mM glucose. In conclusion, the phase modulation of the simple rotator model was sufficient to explain the glucose-dependent changes in the plateau fraction and the U-shaped dominant period of the islet Ca^2+^ oscillations.

### Optimal glucose oscillations can entrain islet Ca^2+^ oscillations

**Fig 3:**
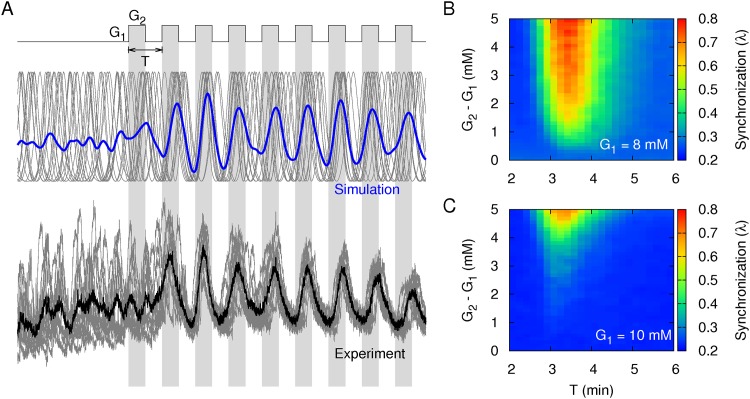
Synchronization of islet Ca^2+^ oscillations under alternating glucose stimuli. (A) Time traces of intracellular Ca^2+^ concentrations within each islet (gray lines) when the glucose concentrations alternated between *G*_1_ = 8 mM and *G*_2_ = 10 mM with a period of *T = 4* min (Top: simulation, Bottom: experiment). The blue line represents the averaged time trace from n = 20 islets (simulation), while the black line represents the averaged time trace from n = 14 islets (experiment). Islets were initially loaded at 3 mM glucose. Before the glucose alternation, islets were equilibrated at 8 mM glucose for 30 min. (B) Synchronization index (*λ*) for various amplitudes (*G*_2_ –*G*_1_) and periods (*T*) of the glucose alternation given *G*_1_ = 8 mM. (C) Synchronization index with *G*_1_ = 10 mM.

The phase modulation of islet Ca^2+^ oscillations by *μ*(*G*) is the response to glucose stimuli. In the present study, we simulated *Ca(t*), which represents the response of the active rotators to glucose concentrations that alternate between *G*_1_ and *G*_2_ with a period of *T* (Fig 3A). For the simulation, we used active rotators for which the intrinsic period and pinning force followed the previously determined distributions, *ω* and *μ*(*G*). Because the rotators experienced common glucose stimuli, the optimal driving signals of the glucose alternation could entrain the rotators to generate synchronous oscillations. For *G*_1_ = 8 mM, we examined the degree of synchronization between the rotators for various amplitudes, *G*_2_ –*G*_1_, and periods, *T* (Fig 3B). The glucose alternation could more effectively provide synchronization when the amplitudes were larger and had an optimal period (3–4 min). We then repeated the same simulation with *G*_1_ = 10 mM, and *μ*(*G*) was less sensitive to the glucose changes than in the simulation using *G*_1_ = 8 mM (Fig 3C). The optimal driving periods did not change, but larger amplitudes were required to induce synchronization.

To confirm these predictions, we measured islet Ca^2+^ oscillations stimulated by alternating glucose concentrations (Fig 3A). Glucose concentrations that alternated between *G*_1_ = 8 mM and *G*_*2*_ = 10 mM with a period of *T* = 4 min could entrain islets to generate synchronous Ca^2+^ oscillations (Table 1). However, glucose alternation with a smaller amplitude (0.5 mM) or with shorter (2.7 min) or longer (5.3 min) periods was not able to entrain islets significantly. We also repeated the same experiment with *G*_*1*_ = 10 mM and confirmed that *G*_*1*_ = 10 mM was less effective than *G*_*1*_ = 8 mM for entraining islets, as predicted in the simulations.

**Table 1:** Alternating glucose stimuli and inter-islet synchronization (*λ*).

Protocol	*G*_*1*_ (mM)	*G*_*2*_ (mM)	*T* (sec)	*λ*	P value	*n*
1	8	8	240	0.33 ± 0.03	-	6
2	8	8.5	240	0.40 ± 0.10	0.11 (1 vs 2)	5
3	8	10	240	0.55 ± 0.12*	0.001 (1 vs 3)	6
4	8	10	160	0.41 ± 0.12	0.06 (3 vs 4)	6
5	8	10	320	0.50 ± 0.11	0.53 (3 vs 5)	6
6	10	10	240	0.36 ± 0.05	-	7
7	10	10.5	240	0.46 ± 0.09	0.03 (6 vs 7)	6
8	10	12	240	0.51 ± 0.16	0.04 (6 vs 8)	8
9	10	12	160	0.36 ± 0.10	0.06 (8 vs 9)	7
10	10	12	320	0.35 ± 0.03	0.03 (8 vs 10)	6

Ten experimental protocols for alternating glucose between *G*_*1*_ and *G*_*2*_ with a period *T* (*n* experiments for each protocol). Given protocols, synchronization between 10 ± 3 islets was quantified by *λ*.

*P<0.01.

### Phase modulation of insulin pulses has a functional role in glucose homeostasis

**Fig 4:**
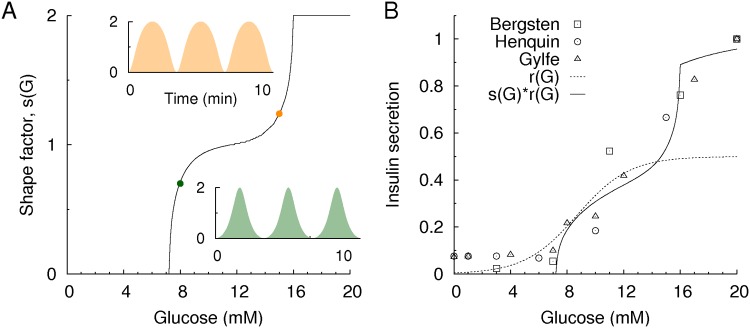
Phase modulation and insulin secretion. (A) Glucose-dependent shape factor, *s*(*G*). Phase-modulated time traces of Ca^2+^ oscillations at 8 mM (green) and 15 mM (orange) glucose. (B) Insulin secretion (solid line), *s*(*G*)⋅*r*(*G*), decomposed into the shape factor, *s*(*G*), and amplitude (dotted line), *r*(*G*). The symbols represent experimental data from Bergsten [41] (square), Henquin [42] (circle), and Gylfe [43] (triangle). The insulin secretion is normalized to have a maximal value at 20 mM glucose.

Thus far, we have examined how islet Ca^2+^ oscillations respond to constant and alternating glucose stimuli. Our ultimate question is whether the phase modulation of the islet Ca^2+^ oscillations has a functional role in glucose regulation. To answer this question, one needs to consider insulin secretion by glucose and glucose regulation by insulin. Given that the intracellular Ca^2+^ concentration is highly correlated with insulin secretion [25, 34], we assumed that insulin pulses have the same phase, *θ*(*t*), as Ca^2+^ oscillations. Therefore, insulin secretion can also be described by a periodic function,

I(t)=r(G)[1+cosθ(t)], (4)

where *r*(*G*) and *θ*(*t*) are the glucose-dependent amplitude and time-varying phase, respectively, of the insulin pulses. Note that *r*(*G*) is the amplitude of the insulin pulses, not the Ca^2+^ oscillations. Thus, the amplitude and the modulated phase of the insulin pulses contribute to insulin secretion. In particular, we defined the latter contribution as a shape factor, *s*(*G*), which is the average insulin secretion with a fixed amplitude, *r* = 1 (Fig 4A). Thus, the average insulin secretion becomes 〈*I*(*G*)〉 = *r*(*G*)⋅*s*(*G*). Given the information of 〈*I*(*G*)〉 and *s*(*G*), we could infer *r*(*G*) (Fig 4B). Notably, 〈*I*(*G*)〉 is most sensitive at *G* = 15 mM, but *r*(*G*) is most sensitive at *G = 8* mM, which is closer to the normal glucose range.

**Fig 5:**
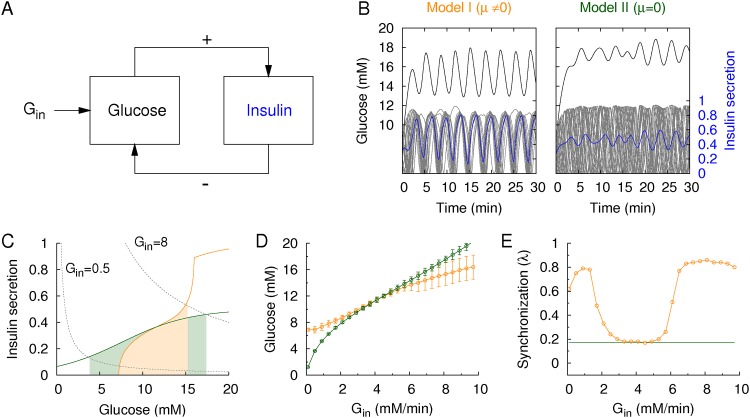
Glucose-insulin regulation. (A) Schematic diagram of the glucose-insulin feedback loop. Infused glucose (*G*_*in*_) increases the glucose concentration. Then, glucose stimulates the islets to secrete insulin, while the integrated insulin decreases glucose by enhancing glucose clearance. (B) Time traces of regulated glucose (black) and insulin secretion (blue) for Model I/II with/without the phase modulation of insulin pulses for *G*_*in*_ = 8mM/min and *v* = 1 min^-1^. Gray traces represent individual insulin pulses (20 islets in the simulation), while blue traces represent their average. (C) Insulin secretion with (orange solid line) and without (green solid line) phase modulation. Dotted lines represent the balance equation between glucose infusion and clearance: *I* = *G*_*in*_ / *vG* with *G*_*in*_ = 0.5 (lower line) and 8 mM/min (upper line) and *v* = 1 min^-1^. The crossing point of insulin secretion and the balance equation determine the stationary glucose concentration. Model I has a narrower range of stationary glucose concentrations. (D) Stationary glucose concentrations and their fluctuations (standard deviation) and (E) synchronization index of insulin pulses for various glucose infusion rates: Model I (orange) and Model II (green).

To complete the glucose-insulin regulation loop (Fig 5A), one also needs to understand glucose regulation by insulin and insulin secretion by glucose. Thus, we model the glucose regulation by insulin,

$$\frac{dG}{dt}=G_{in}-\nu \;I\cdot G,$$ (5)

where *G*_*in*_ is the external glucose infusion rate, and *v* is the glucose clearance rate. The second term in Eq (5) represents the glucose clearance, which is proportional to the insulin level (*I*) and the present glucose concentration (*G*). For the simulation with multiple islets, *I* should be the average insulin secretion of the islets (See Materials and methods).

Now, we have a complete model of glucose-insulin regulation to examine the functional role of the phase modulation of insulin pulses. We compared two models: Model I considers a phase modulation (*μ* ≠ 0), whereas Model II ignores it (*μ* = 0). Thus, Model II always generates pure sinusoidal waves of insulin with a constant shape factor, *s*(*G*) = 1. Given a large glucose infusion (*G*_*in*_ = 8 mM/min), Model I maintained a lower glucose concentration and showed synchronous insulin pulses and an oscillatory glucose concentration, unlike Model II (Fig 5B). Stationary glucose concentrations for *G*_*in*_ could also be mathematically estimated. The stationary condition(*dG* / *dt* = 0) implies that the glucose infusion and clearance are balanced:

$$I=\frac{G_{in}}{\nu \;G}.$$ (6)

In addition to the balance equation, we have another equation, glucose-dependent insulin secretion: 〈*I*(*G*)〉 = *r*(*G*)·*s*(*G*) for Model I and 〈*I*(*G*)〉 = *r*(*G*) for model II due to *s*(*G*) = 1. These two equations uniquely determine the stationary glucose concentrations (Fig 5C). The analysis demonstrated that Model I can maintain the stationary glucose concentration within a narrower range than Model II, given various glucose infusion rates. This result occurs because the shape factor *s*(*G*) effectively modifies insulin secretion by suppressing/enhancing insulin secretion for small/large glucose infusions. We also checked this conclusion with simulations (Fig 5D). In addition to the enhanced glucose regulation, the phase modulation led islets to generate synchronous insulin pulses (Fig 5E). However, when the glucose concentration was raised to a higher level (*G* = 10−12mM) with a large glucose infusion, the synchronization of the insulin pulses decreased because the phase modulation became insensitive to the glucose change occurring at the higher glucose concentration (Fig 2C). This result may explain the diminished insulin pulsatility in diabetic patients with prolonged hyperglycemia.

## Discussion

In this study, we showed that the shape (phase) of islet Ca^2+^ oscillations was modulated depending on glucose concentration. Using the Ca^2+^ oscillation data, we developed a mathematical model that incorporated (i) insulin secretion by glucose and (ii) glucose regulation by insulin. The model demonstrated that the glucose-dependent shape modulation of insulin pulses contributed to tighter regulation of the normal glucose level, compared to their amplitude modulation alone. The additional shape modulation could suppress unnecessary insulin secretion at low glucose, while it could enhance demanding insulin secretion at high glucose. Furthermore, the shape modulation provided a common cue for physically-separate islets to generate synchronous insulin pulses. Otherwise, incoherent insulin pulses from numerous islets would lead to diminished pulsatility of circulating insulin levels. Therefore, we concluded that the shape modulation of insulin pulses was crucial for glucose regulation and inter-islet synchronization.

The diminished pulsatility of circulating insulin levels has been observed in diabetic conditions (refer to a review of [11]). We have found that the shape modulation of insulin pulses was relatively insensitive at high glucose levels (10–14 mM) (Fig 2C). Thus when the external glucose stimulus was alternating between *G*_1_ and *G*_2_, starting from *G*_1_ = 10 mM was less effective to synchronize the islet Ca^2+^ oscillations than starting from *G*_1_ = 8 mM (Fig 3B and 3C). In the glucose-insulin loop simulation, when glucose levels stayed high with large glucose infusion rates (*G*_in_ > 2 mM/min), the inter-islet synchronization was poor (Fig 5E). The asynchronous insulin pulses at hyperglycemic conditions could result in the diminished pulsatility of circulating insulin levels. The incoherent insulin pulses at prolonged hyperglycemia may suggest another reasoning for the loss of insulin pulsatility under diabetes, in addition to the deteriorated pulse generation of individual islets. This mechanism, however, cannot explain the loss of insulin pulsatility for non-diabetic subjects without hyperglycemia [12].

We modeled the phase dynamics of the Ca^2+^ oscillations by adopting the active rotator model instead of describing the detailed molecular processes inside of the cells. Rather than using the constant pinning force (*μ*) of the original active rotator model, we coupled the pinning force *μ*(*G*) to an external stimulus, glucose (*G*). Thus, the simple rotator model could successfully capture the dependence of the plateau fractions and dominant periods of the Ca^2+^ oscillations on the glucose concentration. The modified rotator model is closely related to the theta model (also known as the Ermentrout-Kopell canonical model), which is the simplest model for describing neuronal firing without the details of ionic movement through membranes [35]. A subtle difference is that the external driver is independent of the internal phase in the theta model, whereas the external driver (glucose) is governed by the internal phase (insulin secretion) of the islets in the modified rotator model. Our model is a phenomenological model that drastically simplifies insulin secretion, but precisely incorporates the observed phase modulation of insulin pulses. Therefore, our conclusion would likely be robust, independent of the detailed molecular mechanism of phase modulation. The simple model clearly revealed a link between phase modulation and inter-islet synchronization, and this link might not have been obviously captured in the complex models. The plateau fraction of the Ca^2+^ oscillations was linked to their U-shaped dominant periods of the glucose concentration. A more surprising finding was that the plateau fraction and the U-shaped feature of single islets were linked to synchronization between islets.

To probe target molecules, e.g., voltage-gated Ca^2+^ channel β_3_ subunit in β cells [36], for phase modulation, however, more realistic models are required. An electrophysiological model has been developed to describe the islet Ca^2+^ oscillations and explain inter-islet synchronization [17, 18]. A realistic model will contribute to the formulation of testable hypotheses that apply insights from the simple rotator model. Currently, it is not feasible to noninvasively monitor insulin secretion from multiple islets that experience *in vivo* glucose regulation. Nevertheless, in an *in vitro* experiment, a glucose-insulin feedback loop was constructed using microfluidics, and the experiment demonstrated that the feedback loop could entrain islets to generate synchronous Ca^2+^ oscillations [22].

These wave-like messengers, or hormones, can encode information in their amplitude, frequency, and phase. Hormonal frequency/phase modulation has received less attention than amplitude modulation. The frequency modulation of the luteinizing hormone for reproduction is one rare example [37, 38]. As more hormone measurements at high temporal resolution become available, it is expected that frequency/phase modulation will be more ubiquitously observed in the oscillations of hormones other than insulin.

## Materials and methods

### Experimental animals and islet preparation

All experimental procedures were approved by the Pohang University of Science and Technology Institutional Animal Care and Use Committee (POSTECH IACUC, Korea). C57BL/6J male mice (Jackson Laboratory, Bar Harbor, ME, USA) at 8–12 weeks of age were used. The animals were maintained with a 12 h light/dark cycle with free access to water. Mice were sacrificed by cervical dislocation under anesthesia with CO_2_. Islets were isolated from the pancreas of male C57BL/6J mice (25–30 g) after collagenase digestion. Briefly, 3 mL of 1 mg/mL collagenase P was injected into the pancreas via the common bile duct or by direct injection to expand the pancreas. The pancreas was excised and cut into small pieces, which were then digested with collagenase P to obtain free islets. Islets were then hand-picked under a dissecting microscope and placed in RPMI 1640 containing 11 mM glucose, 10% fetal bovine serum, and 1% penicillin-streptomycin. Islets were cultured at 37°C in a 95% O_2_ and 5% CO_2_ mixture for one day before the experiment.

### Measurements of Ca^2+^ oscillations in islets

To monitor the intracellular concentration of the Ca^2+^ ions, each batch of islets was incubated in a buffer solution containing 3 mM glucose with 2 μM fura-2/AM at 37°C and 5% CO_2_ for 30 min. After incubation, the islets were attached to coverslips using Puramatrix Hydrogel (BD Biosciences, Bedford, MA). The islets were placed in an open perifusion chamber (Live cell instrument, Seoul, South Korea). The chamber was mounted on an inverted fluorescence microscope (IX71, Olympus, Tokyo, Japan) and maintained at 37°C. All of the buffer solutions with various glucose concentrations were delivered to the chambers containing the islets using a fast-flow solution-switching system (VC6; Warner Instruments, Hamden, CT, USA). The flow rate of this system was 3 mL/min, and all of the solutions were maintained at 37°C. The intracellular Ca^2+^ was measured using the 340/380 nm fluorescence ratio with a fluorescence microscope. The microscope was equipped with a 300 W xenon arc lamp (Lambda DG-4, Sutter Instruments, Novato, CA) containing the appropriate filters for excitation of fura-2 at 340 nm and 380 nm. Fluorescence images were acquired with a 100 ms exposure every 1 sec by a CCD camera (ImagEM, Hamamatsu Photonics, Hamamatsu, Japan). The 340/380 nm fluorescence ratios for all islets were obtained and analyzed using MetaFluor software (MDS Analytical Technologies, Sunnyvale, USA). Each islet was defined as a region of interest (ROI). Signals from the ROI were corrected by subtracting the background signal. Islets were perfused within the chamber with 3 mM glucose for 5 min prior to recording the fura-2/AM fluorescence. We used two protocols to measure the islet Ca^2+^ oscillations under constant and alternating glucose conditions. The first protocol started with the glucose concentration at a basal level (3 mM) for 100 sec, followed by an increase to a higher glucose concentration (8, 10, 12, or 15 mM) for 3,000 sec and then a return to the basal glucose concentration for 120 sec. At the end, we depolarized the islets with 25 mM KCl for 250 sec to check their vitality. The second protocol started with the glucose concentration at the basal level for 100 sec, followed by a stimulation with a higher glucose concentration (8 or 10 mM) for 2,000 sec and then alternations of the glucose concentrations between 8 and 8/8.5/10 mM or 10 and 10/10.5/12 mM with a period of 160, 240, or 320 sec. The alternations were repeated 8 times, the glucose concentration returned to the basal level for 120 sec, and finally, the islets were depolarized with 25 mM KCl for 250 sec.

### Chemicals and reagents

Potassium chloride (KCl) and sodium chloride (NaCl) were purchased from Samchun Chemical (Seoul, South Korea). Calcium chloride (CaCl_2_), magnesium chloride (MgCl_2_), 4-(2-hydroxyethyl)-1-piperazineethanesulfonic acid (HEPES), and alpha-D-glucose were supplied by Sigma-Aldrich (Saint Louis, MO). Fetal bovine serum was obtained from Lonza (Walkersville, MD), and penicillin-streptomycin was purchased from Gibco (Carlsbad, CA). Fura-2 acetoxymethyl ester (fura-2/AM) was obtained from Invitrogen (F-1225; Eugene, OR). RPMI 1640 was purchased from Welgene (Seoul, South Korea). Collagenase P (from Clostridium histolyticum; 11213865001) was obtained from Roche Diagnostics (Indianapolis, IN). All of the solutions were prepared using Milli-Q deionized water (Millipore, 18.2 MΩ/cm at 25°C). All of the buffer solutions used in the experiments contained 125 mM NaCl, 5.9 mM KCl, 2.56 mM CaCl_2_, 1.2 mM MgCl_2_, 25 mM HEPES (pH 7.4), and various concentrations of glucose (3–15 mM as indicated). The buffer was supplemented with 1 mg/mL BSA (fraction V; USB 10857; Cleveland, OH).

### Data analysis

We removed a linear trend in the time traces of the Ca^2+^ oscillations to focus on their phase by using the function “detrend” in MATLAB (MathWorks, Natick, MA). This modification set the mean of the time traces to zero. We computed the duration of continuous periods of positive values in the detrended time traces. The time fraction of the positive events was defined as the plateau fraction. We then applied the Fourier transformation to the detrended time traces and obtained their power spectra, and the maximum values defined their peak frequencies. We used the inverse value of the peak frequency as the dominant period. We used the time trace data only if their dominant periods were shorter than 10 min. To measure the degree of synchronization between islets, we adopted a stroboscopic approach that calculates the closeness of the phases between two oscillators [20, 39]. Because the synchronization index (*λ*) was developed to calculate the coherence between two oscillators, we computed *λ* for every pair of islet Ca^2+^ oscillations and defined their average value as our synchronization index [22]. Thus, *λ = 0* and 1 represent complete desynchronization and synchronization, respectively.

### Parameter estimation

We hypothesized that the islets had intrinsic Ca^2+^ oscillations with a predetermined frequency (*ω*), but they generated the oscillations with different frequencies under different glucose conditions due to glucose-dependent phase modulation by *μ*(*G*). The modified frequencies at *G* = 8, 10, 12, and 15 mM glucose became $\omega_{G}=\sqrt{\omega^{2}-\mu_{G}^{2}}$. Assuming that the intrinsic frequency (*ω*) followed a Gaussian distribution with a mean ($\bar{\omega }$) and standard deviation (*δω*), the modified frequency (*ω*_*G*_) should follow a certain distribution, *Q*(*ω*_*G*_). We estimated the likelihood values of $x=(\bar{\omega },\;\delta \omega ,\;\mu_{8},\;\mu_{10},\;\mu_{12},\;\mu_{15})$ that could make *Q*(*ω*_*G*_) closest to the measured distribution, *P*(*ω*_*G*_). To quantify the distance between the two distributions, we used the Kullback-Leibler divergence [40]: *D*(*P || Q*) = Σ_*ω*_*P*(*ω*)log *P*(*ω*)/*Q*(*ω*). We defined a total cost, *C* = Σ_*G*_*D*_*G*_(*P || Q*), for the four glucose conditions. Then, we conducted an importance sampling of *x* with a Monte-Carlo simulation and obtained 10 maximum likelihood values of *x* from ~400,000 samples that produced a lower cost (*C*). Because the parameters were not entirely independent, we set *μ*_*12*_ *=* 0 to fix the scale of *ω* using the evidence that phase modulation was absent in the Ca^2+^ oscillations at 12 mM glucose (plateau fraction = 0.50±0.06). The final estimations obtained from the top 10 likelihood values of *x* were $\bar{\omega }=0.305\pm 0.004$ min^-1^, *δω* = 0.037±0.003 min^-1^, *μ*_8_ = −0.163±0.011 min^-1^, *μ*_*10*_ = −0.028±0.020 min^-1^, *μ*_12_ = 0min^-1^, and *μ*_*15*_ = −0.136±0.018 min^-1^. From these four values of *μ*_*G*_, we obtained the nonlinear function $\mu (G)=\bar{\mu }\text{sinh}[(G-G_{0}^{\mu })/\delta G^{\mu }]$, where $\bar{\mu }=0.016$ min^-1^, $G_{0}^{\mu }=11.6$ mM, *δG*^*μ*^ = 1.2 mM, and the hyperbolic function sinh(*Z*) = (*e*^*Z*^ + *e*^*−Z*^)/2. For the fitting of the nonlinear function, we used the nonlinear least-squares Marquardt-Levenberg algorithm in GNUPLOT. For insulin secretion, we estimated its amplitude from the following published data: Bergsten [41], Henquin [42], and Gylfe [43]. We normalized the three data sets by establishing that insulin secretion became maximal (1 as the dimensionless value) at 20 mM. We hypothesized that the insulin secretion is determined by both the amplitude, *r*(*G*), and the shape factor, *s*(*G*). The phase dynamics determined the shape factor, $s(G)=T^{-1}\int_{0}^{T}Ca(t)dt$. For the amplitude, we assumed a sigmoidal function, $r(G)=0.25[1+\text{tanh}[(G-G_{0}^{r})/\delta G^{r}]]$, where tanh(*Z*) = (*e*^*Z*^−*e*^*−Z*^)/(*e*^*Z*^+*e*^*−Z*^). We then estimated the maximum likelihood values of $G_{0}^{r}=7.6$ mM and *δG*^*r*^ = 8.0 mM that minimized the mismatch, *χ*^*2*^ = Σ_*G*_[〈*I*(*G*)〉−*r*(*G*)·*s*(*G*)]^2^.

### Model simulation

The Ca^2+^ oscillation of the *n*th islet was described by the active rotator model,

$$\frac{d\theta_{n}}{dt}=\omega_{n}-\mu (G)cos\theta_{n}$$

with a Gaussian random number, $\omega_{n}=\bar{\omega }\pm \delta \omega$, and the pinning function, *μ*(*G*). Then, we used *Ca*_*n*_(*t*) = 1+cos* θ*_*n*_(*t*) as the normalized Ca^2+^ concentration to examine its response to constant and alternating glucose concentrations. For the glucose-insulin feedback simulation, we defined the average insulin, $I=N^{-1}\sum_{n=1}^{N}r(G)(1+\text{cos}\theta_{n})$, with an amplitude of *r*(*G*) for insulin secretion. In the simulation, we used *N* = 20 islets. Then, the glucose regulation followed

$$\frac{dG}{dt}=G_{in}-\nu \;I\cdot G.$$

The above *N* + 1 differential equations were numerically integrated using the 2^nd^ order Runge-Kutta method with a sufficiently small time step, Δ*t* = 0.01 [44].

### Statistical analysis

Differences between unpaired observations were evaluated with Student’s *t*-test. The data in the graphs and tables are presented as the means ± standard deviations. Findings were considered statistically significant at P<0.01.
